# Clinical and genetic spectrum of interstitial lung disease in Chinese children associated with surfactant protein C mutations

**DOI:** 10.1186/s13052-019-0710-2

**Published:** 2019-08-28

**Authors:** Da Hong, Dan Dai, Jing Liu, Congcong Zhang, Tingting Jin, Yanyan Shi, Gaoli Jiang, Mei Mei, Libo Wang, Liling Qian

**Affiliations:** 1 Department of Respiratory Medicine, Children’s Hospital of Fudan University, No.399 Wanyuan Road, Shanghai, 201102 China; 2 Children’s Hospital of Fudan University, No.399 Wanyuan Road, Shanghai, 201102 China

**Keywords:** Interstitial lung diseases, Surfactant protein C, Mutation, Chinese

## Abstract

**Background:**

Mutations in the surfactant protein C gene (*SFTPC*) result in interstitial lung disease (ILD). Our objective was to characterize clinical and genetic spectrum of ILD in Chinese children associated with *SFTPC* mutations.

**Methods:**

Six Chinese children with ILD heterozygous for *SFTPC* mutations were included. Candidate genes responsible for surfactant dysfunction were sequenced by next-generation sequencing. Subclones of *SFTPC* with novel mutations were generated and transiently transfected into A549 cells. The functional characterization of mutant surfactant protein C (SP-C) was evaluated by Western blotting and immunofluorescence.

**Results:**

The age of onset ranged from 7 days to 15 months. All cases required supplemental oxygen. Failure to thrive (5/6) was the most significant extra-pulmonary manifestation. Hydroxychloroquine was given as the long-term treatment of lung disease in four patients and two of them responded well. Three mutations were identified in six patients: four with I73T, one with D105G, one with Y113H. Mutations in three patients were inherited and three arised de novo. Western blotting revealed totally different band patterns between mutant SP-C (D105G and Y113H) and the wildtype. Immunofluorescence showed mutant SP-C (D105G) was scarcely trafficked to lamellar bodies but localized well to early endosomes, which was in marked contrast to the wildtype protein.

**Conclusion:**

*SFTPC* mutations were an important cause of childhood ILD in Chinese population. I73T was a common *SFTPC* mutation in Chinese ILD children associated with surfactant protein C mutations.

## Background

Interstitial lung disease (ILD) in children represents a heterogeneous group of respiratory disorders that are characterized by impaired gas exchange and diffuse infiltrates [1]. In children, ILD is most frequently diagnosed in the first year of life with a predominance of genetic entities [2]. In the past decade, significant advances have been made in understanding the underlying causes for childhood ILD (chILD) such as genetic disorders of surfactant dysfunction which result from mutations in genes critical for the function and metabolism of pulmonary surfactant [3]. Among these genes is the surfactant protein C gene (*SFTPC*) located on chromosome 8p21. Its encoding protein, surfactant protein C (SP-C), is a hydrophobic 35-amino-acid polypeptide secreted into the alveolar space by alveolar type II epithelial cells to help reduce surface tension [4]. Since Nogee et al. first reported a case caused by an *SFTPC* mutation in 2001 [5], more than 60 mutations in *SFTPC* have been identified in pediatric ILD patients to date.

Lung disease caused by different *SFTPC* mutations varies greatly, from respiratory distress syndrome (RDS) in neonates to ILD in adults [6, 7]. However, up to now a large proportion of the reported cases are of Caucasian or African descent. Only a few cases with Asian origin were reported [8–10]. Whether patients with different geographic and ethnic origins differ in clinical and genetic spectrum remains unclear. With the increase of awareness of this disease and advances in diagnostic technique, although rare, we discover that *SFTPC* mutations account for a substantial proportion of unexplained ILD with early onset in our Chinese population. Recently, Chen J et al. [11] reported 18 Chinese cases with surfactant dysfunction. Among the 15 patients who had *SFTPC* mutations, 5 different mutations were identified. However, the information regarding the genotype as well as the choice of treatments and therapeutic response of Chinese patients is still limited. Here, we report the clinical features and genetic findings in 6 Chinese subjects heterozygous for *SFTPC* mutations to expand the genetic and clinical spectrum.

## Methods

### Patients

In this study the subjects were identified among symptomatic infants and children who were clinically diagnosed as chILD and suspected of having genetic surfactant dysfunction and then referred for candidate gene sequencing in the laboratory at Children’s Hospital of Fudan University between 2013 and 2018. According to an American Thoracic Society guideline [12], a child is regarded as having chILD if at least three of the following four criteria are present: (1) respiratory symptoms (cough, rapid and/or difficult breathing, or exercise intolerance); (2) respiratory signs (tachypnea, adventitious sounds, retractions, digital clubbing, failure to thrive, or respiratory failure); (3) hypoxemia; and (4) diffuse abnormalities on a chest radiograph or CT scan. Meanwhile, common diseases that can cause ILD were excluded as primary diagnosis by echocardiography and the screening of pathogens, autoimmune antibodies and immune deficiency. Clinical data were collected during the study. This study was approved by the ethics committees of Children’s Hospital of Fudan University. Written informed consent was obtained from all parents or guardians of the patients.

### Genetic analysis

Genomic DNA was isolated from blood of the patients and their parents using the QIAamp DNA Blood Mini kit (Qiagen, Hilden, Germany). Molecular analysis of the disease-causing genes *SFTPB*, *SFTPC*, *ABCA3*, *NKX2–1*, *CSF2RA* and *CSF2RB* were performed through a self-designed gene panel using Ion Torrent PGM (Life Technologies). Targeted genomic regions covered exons and their flanking sequences of these six genes responsible for surfactant dysfunction. Library preparation was conducted by multiplex amplification using the Ion AmpliSeq Library Kit 2.0 (Life Technologies). Sequencing was performed using 316 v2 chips (Life Technologies) on the Ion Torrent PGM platform. We use Torrent Suite software (Life Technologies) to compare base calls. Then we use NextGENe software (SoftGenetics) to read alignments and to call variants with the human reference genome hg19 (NCBI). The variants were then compared with dbSNP. Novel variants were analyzed with in silico tools MutationTaster, SIFT and PolyPhen2.

The validation of the variants was performed by PCR followed by direct Sanger sequencing using 3500XL Genetic Analyzer (Applied Biosystems).

### Functional analysis of *SFTPC* D105G mutation

The methods used to characterize *SFTPC* D105G mutation such as *SFTPC* cDNA expression constructs, A549 cell line transfection, Western blotting and immunofluorescence were described previously [13].

For construction of mutant Flag/SP-C^D105G^, mutagenesis was performed by inverse PCR using KOD Plus Mutagenesis Kit (Toyobo, Japan) with pCDH-EGFP-Flag/SP-C^WT^ serving as a template. The 5′ (forward) primer used for mutagenesis: GCTACCAGCAGCTGCTGATC. The 3′ (reverse) primer: CATACACCACGAGGCCAGTG. All constructs were confirmed by Sanger sequencing.

## Results

### Clinical presentation

From 2013 to 2018, 45 patients were referred for candidate gene sequencing. The age at onset ranged from 0 month to 9 years. Twenty-six were males and 19 females. Six patients (6/45, 13.3%) with heterozygous *SFTPC* mutations were identified (Table 1). One patient (patient 3) has been reported previously [13], but was included into this study because of additional follow-up information. All 6 patients were of Chinese Han origin and born at term with uneventful pregnancy and delivery. There were three males and three females. The age at onset of patients with *SFTPC* mutations ranged from 7 days to 15 months (median 2.5 months) which seems to be earlier than those without surfactant dysfunction (median 1.2 years). There were no difference in symptoms and signs such as cough, tachypnea and cyanosis between patients with and without surfactant dysfunction. However, Failure to thrive was more prevalent in patients with *SFPTC* mutations (5/6) than those without surfactant dysfunction (12/39).

**Table 1:** Clinical presentation of 6 Chinese children with *SFTPC* mutations

Patient	Female/Male	Age at onset	Symptoms	Assisted ventilation	Radiology	Failure to thrive	Other	Outcome
1	F	2 m	Chronic cough Tachypnea Cyanosis	Invasive mechanical ventilation for 1 week Home oxygen therapy at 1.5-2 L·min^− 1^ for 3y	Ground-glass opacity, Interstitial changes	Yes	Clubbed-finger, chest deformity, PH, RCE, laryngomalacia	Alive at 5.5y
2	F	15 m	Chronic cough Tachypnea Cyanosis Respiratory failure	Invasive mechanical ventilation for 6 m at ICU	Bilateral diffuse infiltration, Cyst-like lesions, and Chronic pulmonary fibrosis	No	TSH elevation	Deceased at 22 m
3	F	7d	Chronic cough Tachypnea	Home oxygen(1 L·min^− 1^) for 15 m and intermittent flow for 4 m	Bilateral diffuse infiltration, Ground-glass opacity	Yes	Moderate malnutrition	Alive at 3y
4	M	2 m	Tachypnea	Supplemental oxygen with low flow (1 L·min-1) and intermittent high flow for 6m	Bilateral diffuse infiltration, and interstitial changes	Yes	Severe malnutrition Pectus excavatum	Deceased at 8m
5	M	7m	Chronic cough Tachypnea	Nasal oxygen (0.5-1 L·min-1) for 8m	Ground-glass opacity Interstitial changes	Yes	Severe malnutrition	Alive at 2y
6	M	3m	Tachypnea Cyanosis	Mask oxygen (3.5-4 L·min-1) for 27m	Lung transmittance significantly reduced, Diffuse ground-glass opacity, Scattered subpleural cyst	Yes	Pectus excavatum deformity, Depression in the lower sternum, Moderate malnutrition	Alive at 30m

*PH* Pulmonary hypertension, *RCE* right cardiac enlargement

### Genetic results

Out of all 6 patients, 4 patients carried the hot spot mutation I73T of which two were inherited and two arised de novo (Table 2). Patient 2 was the only one who had a family history of a brother with ILD at 1 year of life. In patient 5, we discovered a once reported mutation D105G [14]. His father and sister who carried the same mutation were asymptomatic. The patient 3 carrying a novel mutation Y113H was described previously [13].

**Table 2:** Genetic information and family history of 6 patients with SFTPC mutations

Patient	Mutation	De *novo* or inherited	SIFT/polyphen2	Family history
1	c.218 T > C, p.I73T	Father I73T	Damaging/damaging	No
2	c.218 T > C, p.I73T	Mother I73T Brother I73T	Damaging/damaging	Yes (brother diagnosed ILD at 1 years)
3	c.337 T > C, p.Y113H	De novo	Damaging/damaging	No
4	c.218 T > C, p.I73T	De novo	Damaging/damaging	No
5	c.314A > G, p.D105G	Father D105G Sister D105G	Damaging/damaging	No
6	c.218 T > C, p.I73T	De novo	Damaging/damaging	No

### Follow-up

Empiric therapy such as corticosteroids, diuretics, antibiotics, aminophylline and cardiac stimulant were used when needed during exacerbations. Hydroxychloroquine was given as the long-term treatment of lung disease in four patients (Table 3). Two of them responded well with an ensuing catch-up of growth and withdrawal of oxygen supply. One (patient 6) responded partially with a decreased demand of oxygen, however weight gain was not remarkable due to repeated diarrhea caused by food allergy. As for the overall outcome, four patients survived with moderate or significant improvement while two died at 8 and 22 months respectively.

**Table 3:** Therapy and follow-up of 6 patients with *SFTPC* mutations

Patient	Presentation at onset	Physical development	Management	Long-term treatment of lung disease (method/ starting age/ ending age)	Improvement	Symptoms at last observation (age)
1	Severe pneumonia Respiratory failure	Normal height Low weight at 7 m (approximate to P3)	Mechanical ventilation Antibiotics Oral prednisone Montelukast Aminophylline Digoxin, Diuretic	Home oxygen therapy	Moderate	Exercise tolerance reduction, Tachypnea after strenuous exercise (5y)
2	Severe pneumonia Respiratory failure	Normal weight (P10–25)	Mechanical ventilation Surfactant Systemic steroids Antibiotics, Diuretic Cardiac stimulant Aminophylline	Persistent mechanical ventilation at ICU (15 m to 22 m until giving up treatment)	No improvement	Recurrent pneumothorax Respiratory failure (22 m died)
3	Pneumonia	Low weight at 9 m (below P3)	Persistent low flow oxygen therapy Antibiotics	Family oxygen therapy(7d to 19 m), HCQ(10 mg·kg^− 1^·d^− 1^ from 13 m to 36 m at present)	Significant (withdrawal of oxygen and weight rises to normol after 6 m HCQ treatment)	Asymptomatic (3y)
4	Severe pneumonia Pectus excavatum	Low weight at 5 m(below P3)	High flow and low flow oxygen inhalation Antibiotics Nasal feeding	Family oxygen therapy (5 m to 8 m), HCQ(10 mg·kg^−1^·d^− 1^ last for a month until dead)	No improvement	Recurrent fever at home Failure to thrive (7 m) Deceased (8 m)
5	Pneumonia	Low height and low weight at 1y(height below P3, and weight far below P3)	Persistent low flow oxygen therapy Antibiotics	Family oxygen therapy (7 m to 15 m), HCQ(10 mg·kg^− 1^·d^− 1^ from 11 m to 24 m at present)	Significant (withdrawal of oxygen after 4 m HCQ treatment), and slight weight gain (P3 at 24 m)	Language retardation Mild dysphagia (24 m)
6	Pneumonia Pectus excavatum Repeated diarrhea (food allergy)	Low weight at 2y (below P3)	Mask oxygen inhalation Antibiotics Nasal feeding	Family oxygen therapy (5 m to 27 m), HCQ (5 mg·kg^−1^·d^−1^ from 24 m for a month then 10 mg·kg^− 1^·d^− 1^ for 5 months at present)	Moderate (Weight gain slightly), Slight decrease in oxygen demand(from initial 3.5–4 L·min^− 1^ to 3 L·min^− 1^ at present)	Increase in food intake Psychomotor retardation (30 m)

*HCQ* Hydroxychloroquine

### Functional analysis of novel mutation

The mutation D105G was only once reported by Willander et al. [14] However, in that report, the mutation carried by two ILD patients was inherited from their asymptomatic parents which was similar to our report. Moreover, no functional data of this mutation is currently available. Therefore, in order to determine the pathogenicity of the mutation, Western blotting and immunofluorescence were performed in A549 cells transfected with wild-type and D105G mutant *SFTPC* expression constructs.

**Fig. 1:**
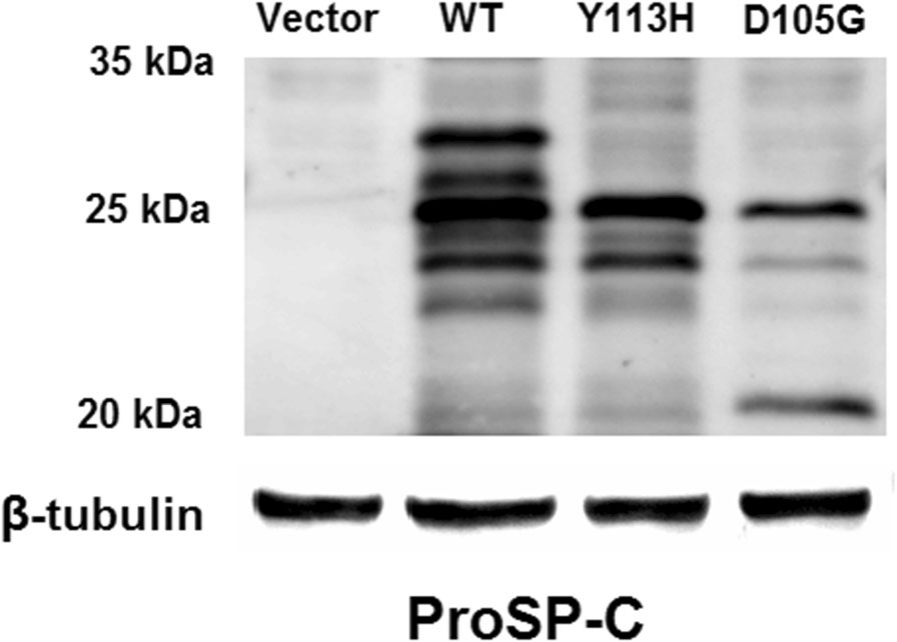
Processing features of ProSP-C^WT^, ProSP-C^Y113H^ and ProSP-C^D105G^

To identify potential processing differences between proSP-C^WT^, proSP-C^Y113H^ and proSP-C^D105G^, lysates of A549 cells transiently transfected with Flag/SP-C^WT^, Flag/SP-C^Y113H^ and Flag/SP-C^D105G^ expression constructs were analyzed by Western blotting. As is shown in Fig. 1, compared with proSP-C^WT^, multiple bands were missing for proSP-C^Y113H^ and proSP-C^D105G^. Meanwhile, an additional band at 20 kDa was observed for proSP-C^D105G^.

**Fig. 2:**
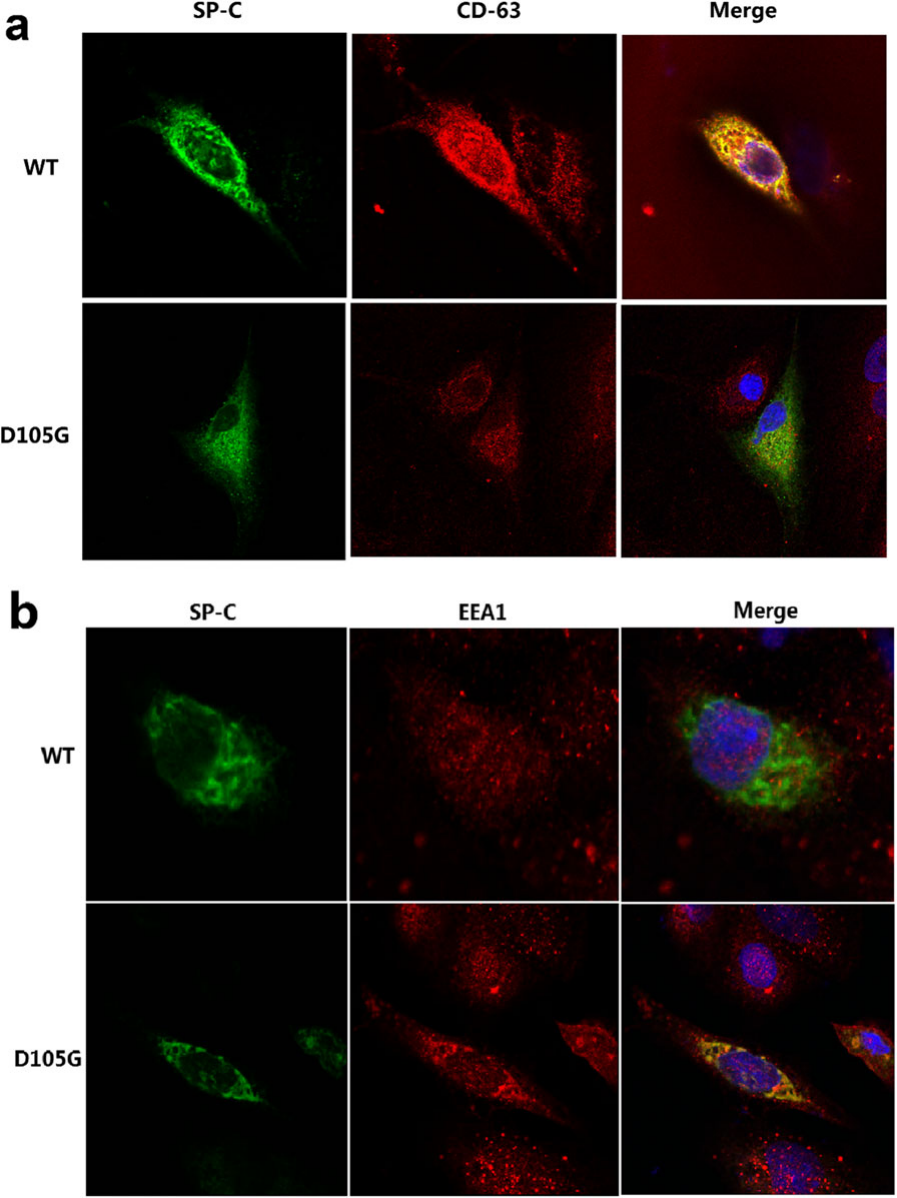
Intracellular localization of ProSP-C^WT^ and ProSP-C^D105G^ forms in transfected A549 cells

Immunofluorescence showed the intracellular localization of proproteins differed between A549 cells expressing proSP-C^WT^ and proSP-C^D105G^ (Fig. 2). ProSP-C^WT^ was localized to CD63 (a marker for lamellar bodies and lysosomes)-positive and EEA1 (a marker for early endosome)-negative vesicles, the expected target vesicle for the wild-type proSP-C. On the contrary, proSP-C^D105G^ hardly colocalized with CD63 but localized well with EEA1 indicating abnormal trafficking and accumulation in early endosomes.

Functioanal analysis of mutation Y113H was described previously [13]. Briefly, in addition to different band patterns revealed by Western blotting as we mentioned above, ultrastructural analysis by transmission electron microscopy showed disorganized cytoplasmic organelles with hollow or eccentrically packed inclusions in cells expressing proSP-C^Y113H^. Immunofluorescence demonstrated that proSP-C^Y113H^ was scarcely trafficked to lamellar bodies but localized well to early endosomes, which was in marked contrast to the wild type protein.

## Discussion

The identification of *SFTPC* mutations has led to significant advances in the diagnosis of interstitial lung disease in infancy and childhood. Due to the lack of diagnostic techniques, patients with SP-C dysfunction were frequently misdiagnosed in the past decades in China. In this study, we described 6 Chinese ILD patients with detailed clinical and genetic information which may help to provide a recognisable pattern for identifying such rare cases in clinical practice.

Most of the patients reported in our study had symptoms within the first year of life and then gradually developed dependence of oxygen with a finding of ground-glass pattern on chest CT. This was consistent with other studies reported by pediatric centers in Western countries [15, 16]. Our study showed an earlier age of onset and a more prevalent failure to thrive in group with *SFTPC* mutations. Referral for genetic analysis should be preferred in ILD patients with these features.

The severity of individuals with *SFTPC* mutations vary greatly, from severe RDS in neonates to mild interstitial lung disease in adults [6, 17, 18]. Clinical outcome at follow-up in our report varied from healthy (age 3 years) to deceased (age 8 and 22 months). Hydroxychloroquine has been reported to improve the clinical status of cases with *SFTPC* mutations. In some case series, 50 to 100% patients responded well to hydroxychloroquine treatment [16, 19, 20]. The exact mechanism of action of hydroxychloroquine is unknown. In addition to having anti-inflammatory properties, hydroxychloroquine has been shown to cause inhibition of the intracellular processing of the precursor of SP-C [21], which may explain its therapeutic effects. 50% (2/4) of our patients responded well to hydroxychloroquine (initiated from 11 months and 13 months of life respectively) and one responded partially (initiated from 24 months of life). However, it should be noted that in the only case not responsive to hydroxychloroquine (initiated from 7 months of life), treatment had just begun for 1 month until he died of pulmonary exacerbation. Nowadays there are still few centers choosing hydroxychloroquine to treat ILD patients resulting from *SFTPC* mutations in China. In the future, more cases and long-term follow-up will be needed to determine the efficacy.

In terms of the genetic findings, we identified 3 different mutations in 6 patients, including two known and a novel mutations. I73T was the most common mutation accounting for 66.7% (4/6) of our patients which was consistent with other literature (28–68%) [15, 20, 22]. Mutations in half of the cases were inherited from parents and only one had family history suggesting imcomplete penetrance. The mechanism of imcomplete penetrance in this disease was still elusive. It was reported that heterozygosity for *ABCA3* (another gene responsible for surfactant dysfunction) mutations modifies the severity of lung disease in individuals with the same *SFTPC* mutation suggesting modifier genes may be involved [23]. In addition, Kaltenborn et al. [24] discovered infection with respiratory syncytial virus potentiated the mutational effects on loss of lung epithelial cell differentiation induced by *ABCA3* mutation. This study indicated that environmental factors such as viral infections may also have a key role in modulating the disease course thus contributing to the phenomenon of imcomplete penetrance.

The mutation D105G identified in patient 5 was once reported [14]. However, the father and sister of the patient who also carried the mutation showed no signs of any lung diseases leading us to questioning the pathogenicity of the mutation. Moreover, no functional data of this mutation is currently available. So together with the novel mutation Y113H, in vitro functional study was performed. According to previous research, many *SFTPC* mutations such as exon 4 deletion cause chronic accumulation of misfolded proSP-C leading to endoplasmic reticulum (ER) stress and alveolar type II cell apoptosis [25, 26]. In our Western bloting analysis, multiple bands of proSP-C^Y113H^ and proSP-C^D105G^ were missing or significantly reduced when compared with the wild-type proprotein suggesting aberrant protein processing of both mutant proteins. However, an accumulated proprotein at 20 kDa was observed for proSP-C^D105G^ while not for proSP-C^Y113H^ indicating distinct proprotein processing. Immunofluorescence assay of transfected A549 cells showed proSP-C^Y113H^ and proSP-C^D105G^ both predominantly colocalized with EEA1 but not with lamellar body marker CD63. So we speculate, unlike proSP-C^WT^ secreted via lamellar body fusion with the plasma membrane and then catabolized mainly by alveolar macrophages, misfolded proSP-C^Y113H^ and proSP-C^D105G^ were endocytosed into endosome. ProSP-C^D105G^ may trigger the unfolded protein response and result in ER stress while proSP-C^Y113H^ may be degraded by the ubiquitin–proteasome system as previously described [13]. The difference in molecular pathogenesis between D105G and Y113H may partially explain why imcomplete penetrance occured in family of patient 5 but not patient 3.

A limitation of the study is that no lung samples of these patients were available. Therefore in vivo data regarding mutant SP-C expression and ultrastructure of alveolar type II cells were unclear. Besides, due to the rareness of this disease, our study is limited by small sample size and further multicenter study will be needed in the future. Also, a longer follow-up period is needed to determine the long-term outcome of the patients and the efficacy of the treatment.

## Conclusions

Our study confirmed *SFTPC* mutations were an important cause of childhood ILD in the Chinese population. I73T was also a common mutation in Chinese ILD children. A part of patients with *SFTPC* mutations can benefit from hydroxychloroquine treatment. A further multi-center longitudinal study will be needed to investigate the phenotype-genotype association and treatment effect in Chinese population.

## Data Availability

The datasets are available from the corresponding author on reasonable request.

## Abbreviations

HCQ: Hydroxychloroquine
ILD: Interstitial lung disease
PH: Pulmonary hypertension
RCE: Right cardiac enlargement
RDS: Respiratory distress syndrome
SFTPC: Surfactant protein C gene
SP-C: Surfactant protein C
